# In Situ Polymerization and Synthesis of UHMWPE/Carbon Fiber Composites

**DOI:** 10.3390/polym17010090

**Published:** 2025-01-01

**Authors:** Elena Fedorenko, Gerrit A. Luinstra

**Affiliations:** Institute for Technical and Macromolecular Chemistry, University of Hamburg, Bundesstraße 45, 20146 Hamburg, Germany; elena.fedorenko@uni-hamburg.de

**Keywords:** fiber-reinforced UHMWPE, in situ ethylene polymerization, carbon-fiber-reinforced thermoplastic composite, material properties

## Abstract

Carbon-fiber-reinforced composites of ultra-high-molecular-weight polyethylene (UHMWPE) are not easily prepared because of their high viscosity, although they can be advantageous in advanced engineering applications due to their superior mechanical properties in combination with their low specific weight and versatility. Short polyacrylonitrile-based carbon-fiber-reinforced UHMWPE composites with fiber contents of 5, 10, and 15 wt.% could easily be prepared using in situ ethylene polymerization. Therefore, MgCl_2_ was generated at the Brønsted acidic groups of the fiber surface by employing a reaction between the co-catalysts Mg(C_4_H_9_)_2_ and AlEt_2_Cl. Titanation with TiCl_4_ resulted in a catalyst directly on the fiber surface. The catalyst polymerized ethylene (2 bar pressure at 50 °C), forming a UHMWPE matrix near the surface; its fragmentation led to polymer particles associated with the fiber. The catalyst activity on the fiber surface of untreated (CF-Pr, 3.48 ± 0.24 wt.%) and oxidized fibers (CF-Ox, 7.41 ± 0.03 wt.%) was 20% lower. CF-Pr compression-molded samples showed tensile strengths of up to 50.4 ± 1.3 MPa, while CF-Ox samples reached 39.1 ± 0.6 MPa, surpassing the performance of composites prepared by melt compounding. The stiffness of 1.58 ± 0.17 GPa for a melt-compounded material was lower than the 3.24 ± 0.10 GPa for CF-Pr and 2.19 ± 0.07 GPa for CF-Ox composites. A fracture examination showed fiber pull-outs, matrix residues on the fibers, and the formation of some extensional polymer fibrils. The latter explains the higher stress at yield and the breakage of the CF-Pr based composites in particular. The potential of in situ polymerized UHMWPE composites for the utilization in high-performance structural applications is thus demonstrated.

## 1. Introduction

The matter of carbon-fiber-reinforced thermoplastics (CFRTPs) is a timely and highly significant subject in both scientific and social contexts. Applications in transportation, aerospace, and sports goods are expected to reach a compound annual growth rate of 7.8% in the timespan of 2022 to 2030 [1]. Drivers for the development are, for example, regulatory pressure (e.g., to increase fuel efficiency and the adoption of new emissions regulations in the automotive sector) or economics, like the implementation of effective manufacturing techniques. This goes along with the redesign of materials to introduce better recyclable, more cost-efficient, and, thus, “higher”-performance materials: carbon-fiber-reinforced thermoplastics provide a lightweight and easily processable, weldable material solution without compromising the high impact resistance, albeit with some restraints with respect to fatigue (cold flow) [2]. The combination of high stiffness at low density allows for significant weight savings.

A straightforward process that gives control over the material properties by adjusting the filler content and matrix properties for a specific application should be of interest in composite material usage. Accordingly, elaborating on an advancement in the corresponding engineering and/or chemical science seemed appropriate. The most used matrix polymers in the context above are thermosets. Their moderate recyclability, reparability, and long cycle time in production are apt for improvement. Thermoplastic matrix materials, such as polyethylene, offer a certain recyclability as material and the option for thermo-reshaping, making them key components with superior life cycle analyses, fit for the realm of circular economy materials.

Ultra-high-molecular-weight polyethylene (UHMWPE) is (along with a prechosen set of material properties) perhaps the thermoplastic material with the most extensive profile, including its low pricing. It is understood as a linear polyethylene with a molecular weight of 3000 kg/mol or higher [3]. Chemical resistance, a low friction coefficient, high impact strength, and biocompatibility make the material attractive for variable uses in the automotive, aerospace, recreation, and medical sectors [4]. Despite its exceptional properties, UHMWPE has the major drawback of an extremely high melt viscosity [5]. Conventional HDPE-processing techniques, such as injection molding and extrusion processing, are not very suitable for UHMWPE [6]; rather, thermal processing involves ram extrusion [7]. It remains difficult to prepare parts with small features for the same reasons. Thermal sintering is another way of dry-processing UHMWPE powders: it also has limits, but perhaps with the option of easier improvement [8].

The property profile of UHMWPE, in particular, the Young’s modulus, can still be enhanced by generating a composite with carbon fibers [9]. The manufacturing process of CFRTP with a separate UHMWPE matrix requires more than just high temperature and high pressure to ensure the sufficient impregnation of the textile fiber with the highly viscous thermoplastic melt. Preparation methods for UHMWPE/CF composites include polymer solution preparation [10,11], (hot) ball-milling processes [9,12], co-weaving [13], and the direct dry mixing and compression molding of fiber with UHMWPE powder [4,14,15]. The latter process would benefit from a predispersion of matrix and fiber.

Although solution, compression, and sintering processes are being explored as mechanical manufacturing processes for the production of UHMWPE/CF composites [9,15], conventional composite production processes have been found largely unsuitable for the UHMWPE matrix polymer. This is because it is difficult to ensure a homogeneous distribution of the polymer matrix without extreme conditions, the use of (excessive amounts of) chlorinated solvents, or the overstressing of the polymer and/or carbon fibers. A good dispersion of fibers is, however, of eminent importance to the performance of the composite. Additionally, it is important to maintain the continuity of the matrix/fiber interface to ensure load transfer from the polymer to the fibers [16].

The high viscosity of a polymer matrix and the characteristics of the carbon fiber surface often lead to insufficient penetrability and the incomplete embedding of the fiber in many methods, ultimately resulting in a decline in the load resistance of the composite material [17,18]. The weak interfacial bonding between carbon fiber and polyethylene matrix polymer caused by the low wettability of the carbon fiber underlies the unsatisfactory mechanical performance in part. The oxidation of carbon fiber is generally employed to improve matrix fiber interactions, with a moderate impact in the case of polyolefins [10,19,20]. Furthermore, the higher process temperature and pressure required for composite preparation may cause polyethylene degradation that could negatively impact the properties of the final material [21]. Only with careful consideration and expert handling can these challenges be (partly) overcome to produce a high-quality composite material.

In situ ethylene polymerization, i.e., synthesizing the polymer matrix on the fiber surface by generating polymerization active sites directly on its surface, eliminates some of the disadvantages mentioned above and can also shorten process chains. The in situ method enables fast manufacturability since no curing time, e.g., for crosslinking reactions, is required, and the intermediate materials have basically an unlimited shelf life. In situ polymerization methods encapsulate individual carbon fibers during matrix generation, so that no further mixing processes are required. In the case of polyethylene, the matrix may be partially disentangled, leading to lower initial viscosities at thermal processing during which entanglements build up, which is of particular importance for UHMWPE [22].

Polyethylene carbon composites were also prepared in the past by in situ polymerization with carbon nanofillers such as carbon nanotubes [23,24,25,26] or graphite [27]. These studies primarily focused on reaching an excellent filler distribution by utilizing the low viscosity of the polymerization medium for the dispersion of the filler. The in situ generation of the matrix polymer may also result in an enhanced wetting. The immobilization of the metallocene/MAO catalyst on macroscopic glass fiber and the in situ polymerization of propylene achieved composites with an improved fiber matrix adhesion [28]. The preparation of polyamide [29] and poly(methyl methacrylate) [30] thermoplastic composites by the in situ method was also successful, but the corresponding composites exhibit inferior chemical resistance and durability relative to polyethylene-based composites. In situ polymerization more generally is certainly a versatile approach, also with potential applications in the preparation of biobased and/or biodegradable composite materials. It has also been explored in the preparation of biocomposites, like PLA/cellulose nanocrystals [31] and PS/wood [32], demonstrating its relevance in the advancement of eco-friendly and high-performance materials.

The preparation of UHMWPE/CF composites by in situ polymerization has not received much attention this far, although it has a high potential for simplifying the application and the broadening of the property profile: Encapsulations with UHMWPE by the in situ polymerization of zirconia particles [33], diamond nanoparticles [34], and carbon nanoparticles [35,36] have been shown to improve tribological behavior, conductivity, and mechanical properties, and simplify composite processability. The primary objective of this work is to demonstrate the features and advantages of in situ polymerization for preparing UHMWPE/CF composites, utilizing a highly active and easily accessible “quasi-supported” Ziegler–Natta catalyst at the fiber surface. The catalyst support is previously anchored to the fiber surface in contrast to the usual catalyst anchoring with the result of reaching a broad product distribution [37]. Overall, a medium-sized semi-finished part or composites with a high polymer content was created. The material can be processed into a ready-to-use component through compression molding, establishing a foundation for the scalable production of high-performance, process-ready composite materials.

## 2. Materials and Methods

### 2.1. General Procedures and Sources

All experiments followed the standard Schlenk technique under a dry nitrogen atmosphere (grade 4.5, Linde AG, Hamburg, Germany). Ethylene (grade 3.5, Gerling Holz & Co., Hamburg, Germany) underwent purification over columns with molecular sieve (4 Å) and BASF catalyst R3-11/G. Toluene (99.9% purity, Merck, Darmstadt, Germany) was purified by distillation and passage through similar columns as in ethylene purification. TiCl_4_ (1 M toluene solution), Al(C_2_H_5_)_2_Cl (1 M toluene solution), and Mg(C_4_H_9_)_2_ (1 M heptane solution) were procured from Sigma-Aldrich (Taufkirchen, Germany) and used without additional purification. PAN-based Tenax^®^ 5001 N carbon fiber (8 µm diameter, aspect ratio of 250, CF-Pr) was provided by Leibniz-Institut für Polymerforschung Dresden, Germany. HNO_3_ (65%, Merck, Darmstadt, Germany) for the wet oxidation process of the carbon fiber was used as received.

### 2.2. Surface Oxidation of Carbon Fiber

Non-sized PAN-based carbon fibers (CF-Pr) were subjected to modification through wet oxidation by immersing them in concentrated HNO_3_ (65%) in a boiling flask and keeping them at 115 °C under reflux for 60 min. Subsequently, the oxidized fibers (CF-Ox) underwent washing with demineralized water and extraction with demineralized water in a Soxhlet extractor for 24 h. The resulting oxidized fibers were then dried at room temperature in vacuo for 48 h.

### 2.3. Functionalization of Carbon Fiber with Ziegler–Natta Catalyst Species

A 100 g sample of carbon fiber was loaded into a Schlenk flask and dried under dynamic vacuum at 80 °C for 24 h. After cooling to 50 °C, the flask was filled with nitrogen, and 50 mL of toluene were added. Mg(C_4_H_9_)_2_ (0.1 mmol) was introduced and stirred for 30 min. Subsequently, AlEt_2_Cl ([Al]:[Mg] = 6) and TiCl_4_ ([Mg]:[Ti] = 500) were added, and stirred for 30 min prior to filtration and washing with toluene twice. The resulting carbon fiber/Ziegler–Natta catalyst composites were then dried in vacuo at room temperature for 48 h.

### 2.4. In Situ Synthesis of Carbon-Fiber–PE Composites

All reactions were conducted in a 1 L glass reactor (Büchi Glas Uster AG, Uster, Switzerland) equipped with a diagonal-blade steel stirrer. CF-Pr or CF-Ox were loaded into the reactor and degassed at 80 C under dynamic vacuum for 2 h. The reactor was then flushed with nitrogen and cooled down to 50 °C polymerization temperature and charged with 300 mL toluene. A standard reaction involved an addition of Mg(C_4_H_9_)_2_ (0.85 mmol/g_CF_) with immobilization time of 30 min while stirring at 300 rpm. Then, AlEt_2_Cl ([Al]:[Mg] = 6) and TiCl_4_ ([Mg]:[Ti] = 500) were sequentially added. After 30 min, ethylene saturation at 2 bar partial pressure initiated the reaction, lasting 5–50 min based on desired filler content. Ethylene volume steam was limited at 500 mL/min using mass flow controller (Brooks Instruments, Veenendaal, The Netherlands, 5850TR series). The reaction was quenched by releasing the pressure and addition of ethanol (20 mL). The composites were filtered, thoroughly washed with ethanol and acidified water (7%), and dried in vacuo at 40 °C for 48 h.

### 2.5. Characterization

#### 2.5.1. SEM EDX

Composite morphology was investigated using scanning electron microscopy (SEM, Carl Zeiss Gemini Leo 1525 field emission microscope, Peine, Germany). Energy-dispersive X-ray spectroscopy in combination with SEM was used to assess catalyst compound remains on carbon fiber. EDX data analysis was conducted at 20 kV electron acceleration using an Octane plus silicon drift detector. Samples were coated with platin prior to measurement. Atomic composition of one sample was measured in three spots prior mean value formation.

#### 2.5.2. Thermogravimetric Analysis

The carbon fiber content of the composite was determined by utilizing Mettler Toledo TGA1, Giesen Germany. The measurements were run using heating rate at 20 K/min from 25 °C to 800 °C under 20 mL/min air flow. The filler content was taken from the calculation from the residual masses at 800 °C.

#### 2.5.3. Differential Scanning Calorimetry

Melting temperature, *T*_m_, and crystallization temperature, *T*_c_, was determined by differential scanning calorimetry (DSC) using DSC1 (Mettler–Toledo, Giesen, Germany). Data were collected using the first heating stage (rate of 10 °C/min, range from 25 to 200 °C) and normalized by the UHMWPE content under correction of the fiber content.

#### 2.5.4. Size Exclusion Chromatography (SEC)

Molecular weight distribution of the polymeric matrix of the composites was determined by size exclusion chromatography (SEC) on a PL-GPC 220 (Schönwalde-Glien, Germany) in 1,2,4-trichlorobenzene as an eluent with a flow rate at 1 mL/min at 135 °C. The signals were detected by reflective index detector and evaluated using polystyrene reference calibration.

#### 2.5.5. Tensile Testing

The specimens for tensile testing were fabricated through heat-pressing/compression-molding virgin composites in a mold (100 × 100 × 2 mm) at 190 °C and 200 bar pressure for 10 min. Melt-compounded samples were prepared by dry-mixing and pressing in the molten state at 190 °C and 200 bar pressure for 1 min. These were folded afterwards and repeated ten times. Following cooling to room temperature, the samples were prepared by die-cutting in accordance with DIN EN ISO 527-5A [38]. Tensile testing was conducted on a Zwick Roell Zwicki-LineZ1.0 machine (Zwick GmbH & Co. KG, Ulm, Germany) under room temperature with a 1 kN force transducer, following DIN EN ISO 527 at a rate of 5 mm/min [39]. The Young’s modulus is calculated by determining the slope of the stress–strain curve at low deformations where the load initially is transferred from the matrix to the fiber ends [16], hindering the plastic flow of the polymer matrix.

#### 2.5.6. Hardness Testing

Shore D hardness measurements were performed as per DIN EN ISO 868 standard [40] under room temperature with a Shore D durometer Sauter TI TH-D (Sauter GmbH, Balingen, Germany). A dwell time of 10 s at 5 spots of each heat-pressed specimen was applied to measure the hardness.

## 3. Results and Discussion

**Figure 1:**
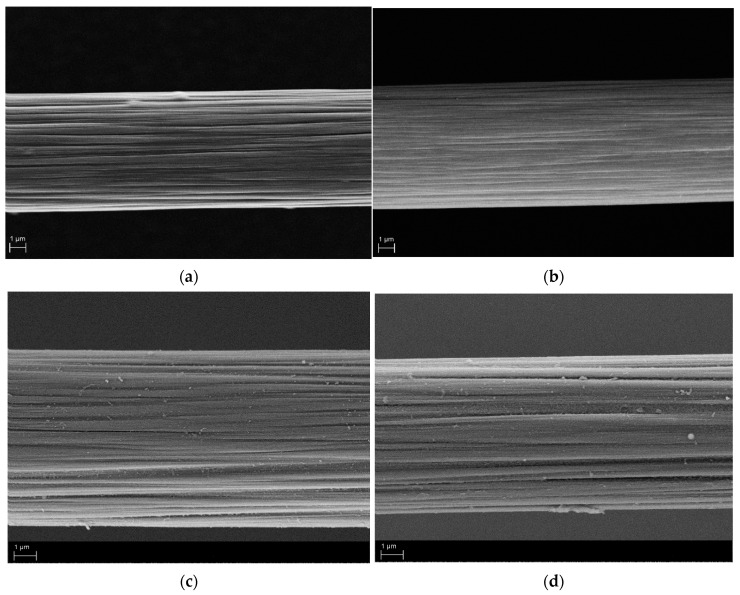
SEM image of CF-Pr (**a**) and CF-Ox (**b**), resp., after treatment with TiCl_4_–AlEt_2_Cl/Mg(C_4_H_9_)_2_ CF-Pr (**c**) and CF-Ox (**d**).

The (pristine) carbon fibers (CF-Pr) chosen for this study have a surface oxygen content of about 3.5% (Figure 1a). The oxygen groups on carbon fibers are known to result from deformations during the manufacturing process [41,42]. These fibers were also subjected to wet oxidation to increase the surface defects and oxygen content. It was anticipated that polar entities would be of importance for the anchoring of the polar catalyst components to the otherwise rather inert carbon [25]. Carbon fiber functionalization was achieved through wet oxidation (CF-Ox) with concentrated nitric acid at reflux for 60 min (Figure 1b, Table 1) [9,43].

### 3.1. Generation of Carbon-Fiber Ziegler–Natta Catalyst

A highly active MgCl_2_-supported Ziegler–Natta catalyst was generated on the surface of CF-Pr and CF-Ox by an in situ catalyst-forming method (Figure 1c,d). It is based on the known combination of Al(C_2_H_5_)_2_Cl and Mg(C_4_H_9_)_2_ which forms in situ Lewis acidic MgCl_2_ precipitates and AlEt_2_Bu (Equation (1)) [25,44,45]. The polymerization active species is formed by the absorption of TiCl_4_ on the MgCl_2_ surface, followed by alkylation and tentatively cationization (Equation (2)) [44,45]. The TiCl_4_-AlEt_2_Cl/Mg(C_4_H_9_)_2_ catalyst system demonstrated significant activity in ethylene polymerization [45]. The high activity of the TiCl_4_-AlEt_2_Cl/Mg(C_4_H_9_)_2_ catalyst results from the fine distribution of a high number of small particles [46]. It may be classified as a pseudo-homogeneous catalyst, making it a suitable choice for reaching the goal of this study: each component is soluble in hydrocarbons independently; yet, their combination leads to the formation of finely dispersed quasi-colloidal solids, with the option of linking to surfaces [45].

(1) $${\mathrm{Mg}\left(C_{4}H_{9}\right)}_{2}+2\mathrm{Al}(C_{2}H_{5}{)}_{2}\mathrm{Cl}\;\to [\mathrm{Mg}{\mathrm{Cl}}_{2}]+2\mathrm{Al}(C_{2}H_{5}{)}_{2}(C_{4}H_{9})$$

(2) $$[\mathrm{Mg}{\mathrm{Cl}}_{2}]+\mathrm{TiC}l_{4}+\mathrm{Al}R_{3}\to \;[{\mathrm{Cl}}_{3}{\mathrm{Ti}}^{+}{\left(R)\right]}_{\mathrm{ads}}/[\mathrm{Mg}{\mathrm{Cl}}_{2}]\;\mathrm{Al}R_{3}{\mathrm{Cl}}^{-}\;(R=Et,Bu)$$

**Scheme 1:**
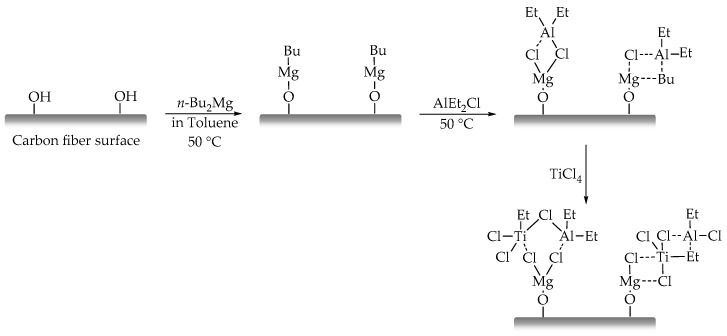
Immobilization of carbon fiber with TiCl_4_–AlEt_2_Cl/Mg(C_4_H_9_)_2_ Ziegler–Natta catalyst.

Thus, CF-Pr and CF-Ox were firstly treated with Mg(C_4_H_9_)_2_ (1 mmol/g_CF_) in toluene. It is envisioned that its hydrolysis by hydroxyl entities at the fiber surface will lead to “BuMg-O” anchor sites (Scheme 1). The hydrolysis reaction is usually highly efficient, like reported in analogous investigations, e.g., in one involving the reaction of silica with an allyl magnesium reagents [47,48]. Subsequently, AlEt_2_Cl ([Al]:[Mg] = 6) and TiCl_4_ ([Mg]:[Ti] = 500) were added in that order. The addition of AlEt_2_Cl leads the in situ formation of Lewis acidic Cl-Mg-Cl entities, some of which may be connected to Mg-O-CF by a chloride bridge, forming small crystals. The polymerization active complex is formulated as a double-Cl-bridged Ti-Mg- and Al-coordinating complex at the crystal surface [45,49,50]. This class of catalyst represents a typical heterogeneous catalyst system, but are here connected to another (i.e., fiber) surface.

SEM images of the fiber with deactivated catalysts reveal the perceptible remains of the hydrolyzed catalyst system of Mg/Al/Ti clusters (Figure 1c,d). The EDX analysis of CF-Pr and CF-Ox indicate no significant difference in the loading of the elements of the catalyst components (Table 1). Note that the higher oxygen surface content is interpreted as the result of the hydrolysis of the catalyst after contact with the surrounding moist air.

### 3.2. Synthesis, Morphology, and Characteristics of Polymer and Composites

A reference polyethylene was synthesized using identical catalyst components, ratios, and procedures (to give TiCl_4_–AlEt_2_Cl/Mg(C_4_H_9_)_2_ in toluene slurry) and ethylene polymerization at 50 °C. Thus, the [Al]/[Ti] molar ratio was set at 500, and the [Al]/[Mg] = 6 with a total Mg content of 0.57 mmol/L. The polymerization was carried out at 2 bar of ethylene pressure in a semibatch operation (continuous dosing of ethylene). The polymerization activity is high at about 7 t_PE_/mol_Ti_·h (Table 2, Figure S1 (S#: c.f. Suppl.)). The product had a weight average molecular weight 5.10 × 10^6^ Da and a polydispersity index (PDI) of 6.5. The broader distribution is typical for the action of this heterogenous catalyst system, with, tentatively, several types of active centers [45]. The product could, as expected, not be processed by conventional melt processing in an extruder.

**Figure 2:**
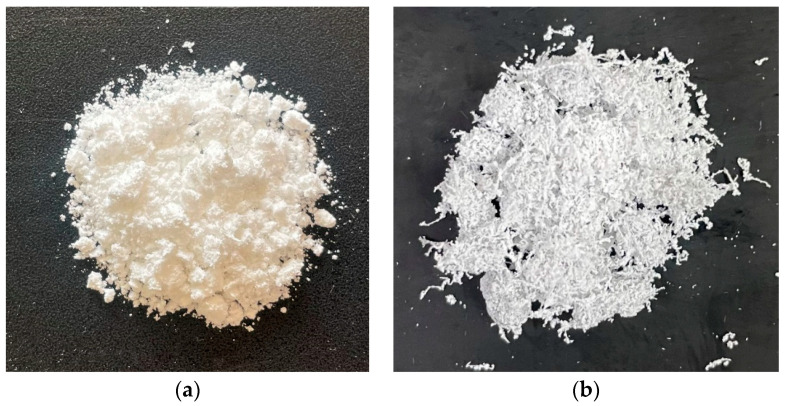
Morphology of prepared neat UHMWPE (**a**) and a UHMWPE/CF-Pr composite (**b**).

The (for this catalyst system) unusual high molecular weight of the product is attributed to the relatively low TiCl_4_ concentration (Ti = 6.7 μmol/L) in the reactor [45]. This gives a high ethylene:Ti ratio, which may be the decisive factor for the chain growth to the region of UHMWPE (Figure 2a). Termination is a rare event as, formally, only about three chains are formed per titanium atom (Table 2). This UHMWPE was used as a reference material and as a matrix for composites prepared by manually mixing it with CF and the subsequent hot compression molding.

**Figure 3:**
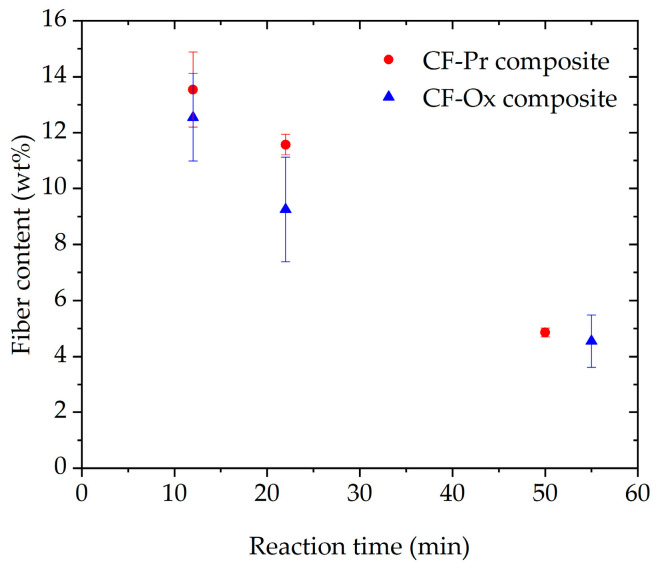
Fiber content of CF-Pr and CF-Ox dependent on the polymerization reaction time.

UHMWPE/carbon fiber composites were generated accordingly. Therefore, the CFs were thoroughly dried and treated with 0.85 mmol/g_CF_ of Mg(C_4_H_9_)_2_. AlEt_2_Cl ([Al]/[Mg] = 6) and TiCl_4_ ([Al]/[Ti] = 500) were again added after 30 min. The heterogenous catalyst is formed in the latter period, both on the carbon fibers and in the surrounding solution. After another 30 min, ethylene was submitted to the reactor at a set pressure of 2 bar. A mass flow controller was used to monitor the amount of ethylene passed into the reactor. The polymerization time was then adjusted to effectively control the monomer amount to reach composites with CF percentages of approximately 5, 10, and 15 wt% (the ratios were calculated post-polymerization from the yield, and the values were supported by the TGA of the products) for both the pristine and oxidized CF derivatives (Figure 3).

**Figure 4:**
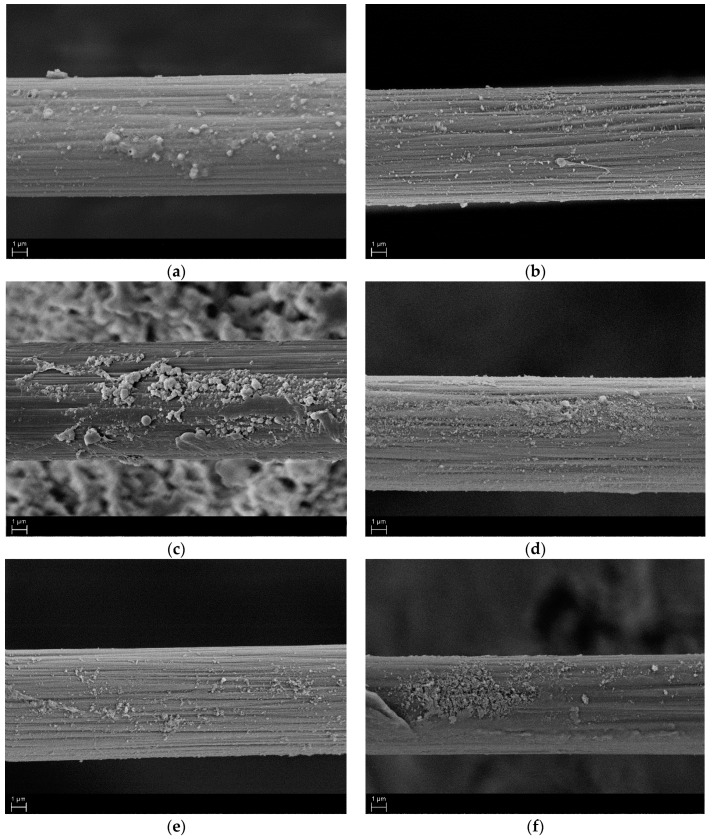
SEM images of CF-Pr fibers after in situ polymerization at fiber content of 5 wt% (**a**), 10 wt% (**c**), and 15 wt% (**e**), and SEM images of CF-Ox after in situ polymerization at fiber content of 5 wt% (**b**), 10 wt% (**d**), and 15 wt% (**f**).

The products appear as white solids, with no visual trace of the black fibers left (Figure 2b). The fibers (or, better, the fiber bundles) appear completely imbedded in polyethylene (Figure S2). The produced composites have a morphology, reminiscent of “turnings”. They are readily isolated from the reactor by collection on a frit. The constitution of the product indicates that the polyethylene is formed in the close vicinity of the fibers with the catalyst separating (in part) from the fiber bundles, most probably by the usual catalyst fragmentation [51]. The fiber-associated porous particulates of UHMWPE are loosely bonded to each other and to the fiber surface. It thus seems likely that the catalyst particles initially were concentrated around the fiber anchor points or to the catalyst particles covalently bonded to the surface through polar interactions (Scheme 1). The formation of the “raspberry” morphology of the replica of loosely agglomerated catalyst (precursor) particles is also easily recognized in SEM images of the composites from the reactor (Figure S2). The fibers appear mostly with their surface (and sizing; Figure 4) exposed with localized conglomerations of polyethylene. In accordance, the absence of reactor fouling suggests that the polymer was predominantly formed on the surface of the catalyst.

SEM images of the composite fiber surfaces are indicative of the formation of only a small amount of polyethylene at the surface and in the grooves of the CFs (Figure 4). A distinct coverage is recognizable in form of a thin layer (like a veil) next to some polymer crystallites. The generation of polyethylene at/near the surface thus seems to result in some kind of a sizing of unknown composition and adhesion quality; an examination suggests that at least some polyethylene is adhered to the surface (vide infra), but catalyst system residues may also be present. There is no obvious difference between the appearance of the fibers dependent on the polymerization reaction time nor dependent on the surface oxygen content of the fiber.

The catalyst activity is somewhat lower in the presence of CF-Pr than in the synthesis of the reference UHMWPE, and even more so in the case of CF-Ox (Table 2). A part of the dibutyl magnesium will react with the hydroxyl entities at the fiber surface (Scheme 1) under the loss of butane. This reaction will reduce the amount of MgCl_2_ that can be formed, and some more AlEt_2_Cl may remain unreacted. At the same time, the part of the catalyst associated with the surface of the fibers will be less accessible. The clustering of the main part of the catalyst particles to the fiber confines it to a smaller volume where higher local concentrations of catalyst may lead to a local lower ethylene concentration (like in a diffusion-controlled polymerization). The clustering is presumed because of the morphology of the product. The higher local catalyst concentration may also lead to more entangled polyethylene, which may constitute a somewhat higher barrier for ethylene to diffuse to the catalyst surface [52,53,54,55].

The activity of the catalyst also decreases with reaction time, which is expected in such a case (Figure S1). Composites prepared in a shorter timespan with a fiber content of 15 wt%, and, thus, the lowest UHMWPE content, would have active polymerization centers that are not as much encapsulated by the formed polymer, resulting in higher ethylene consumption rates. These 15 wt% composites can be regarded as forerunners of composites with a lower fiber content. Catalyst activities in that phase indeed are close to that in the polymerization to the reference UHMWPE; however, the number of terminations appear much lower. In accordance, the PDI of the matrix UHWMPE CF-Pr composite is much lower than that of the reference (Table 2).

The narrower distribution of the early stages with formally fewer chains per titanium indicates a more uniform polymer formation. This would be consistent with a chain transfer to aluminum as the major termination reaction, which would be slower when the catalyst is confined to a smaller volume (for a diffusion limited rate: the type of chain termination, however, is not elucidated) [56,57]. The matrix obtained in the presence of CF-Ox is more influenced by terminations, leading to lower molecular weights and also a broader distribution. The higher concentration of hydroxyl at the fiber surface may have led to a finer dispersion of the catalyst particles along the fiber, which may be more like the situation in the preparation of the reference UHMWPE with more terminations.

The weight average molecular mass of the matrix polymer in the composites increases slightly with the reaction time, consistent with an almost living polymerization process (Table 2). The distribution widens in the reaction timespan leading to the 10 wt% composites: more terminations take place as the polymerization reaction rate is lower in a transition phase, but the molecular mass still increases effectively, which is characteristic of the final stage of the polymerization. In that last stage, leading to the 5 wt% composites, the distribution again narrows apparently because of fewer terminations and thus with a further increase in the molecular weight. The PDI of the matrix of the composites with 5 wt% of filler end up being the lowest in the respective series. Termination in the later phase seems to subside (possible after the full conversion of small aluminum alkyls) [56,57,58], and the later-formed chains are dominating the ensemble with their much higher mass.

### 3.3. Thermal Properties and Shore Hardness

**Figure 5:**
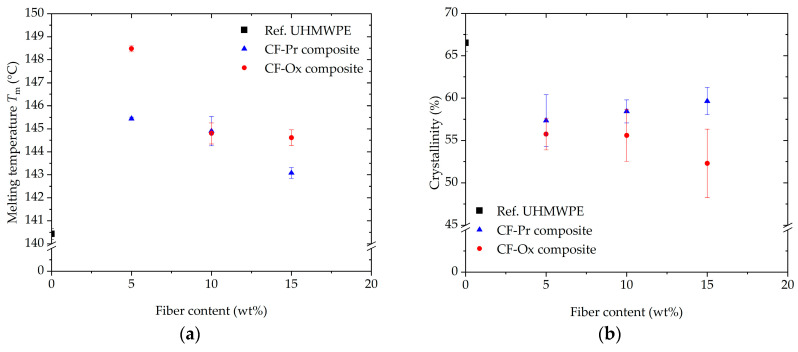
Melting temperature *T*_m_ (**a**) and degree of crystallization (**b**) of the first heating scan of the reference UHMWPE, UHMWPE/CF-Pr, and UHMWPE/CF-Ox composites.

The melting behavior in the first heating scan contains some information on the polymer formation; the differences in the products can be understood along the polymerization behavior suggested above. The polymeric products show the typical high melting points of UHMWPEs for the entangled UHMWPE [59]. The melting point of the virgin composite matrix UHMWPEs is up to eight degrees higher than that of the reference UHMWPE with a *T*_m_ of 140.4 °C (Table 2, Figure 5a). The catalyst system without the presence of CF is more active in the better distributed (more dilute) conditions of its preparation. The faster formation of polyethylene results in a higher concentration of backbone segments and a faster crystallization with more nucleation. This leads to smaller crystallites, with the part of smaller-molecular-mass chains in the broader distribution also promoting fast crystallization.

The higher melting points of the composites go along with a lower overall initial crystallinity, which, in part, is substantially lower (5 to 15%) than that of the reference polymer (Figure 5b). Apparently, larger crystallites are formed at the lower rate of polymerization, progressively in the presence of ever more polyethylene at longer reaction times. It is possible that the crystals are formed with fewer entanglements, which could limit the segment addition to the already formed lamellae. Larger crystals would also explain the overall lower crystallinity. The topological limitation of the polymer chain segments to add to crystal surfaces increases the amorphous part [60,61]. In accordance, the longer the polymerization time (leading to the lower CF content), the higher the melting point of the virgin matrix polyethylene.

The melting range of the composites are also broader than that of the reference UHMWPE. The DSC trace of the reference UHMWPE shows only a small shoulder at a higher temperature (Figure S3), indicating a low content of separately formed larger crystals, possible when major diffusion barriers start to arise [62,63]. The higher dispersion of the catalyst centers is favorable for a fast initial polyethylene formation.

In contrast, the morphology of the composites indicates much more localized polyethylene formation, which tentatively goes along with higher diffusion barriers. The ethylene polymerization process goes through several different stages during composite formation: the polymerization time is longer as the activity is lower, and, as reaction time is longer, more higher-melting PE is formed, which shifts the maximum of the DSC trace to higher temperatures [64]. The peak width increases accordingly with the reaction time, as domains with different polyethylenes with respect to molecular mass and, thus, crystallinities are formed.

The crystallinity and the melting temperature of the CF-Pr composites indeed have the expected opposing trend (Figure 5a,b). The larger the crystallites are, the more amorphous polymer remains. A more complex situation arises for the CF-Ox composites, wherein the overall crystallinity and melting point both increase with polymerization time, i.e., with decreasing fiber content. The crystallinity of the CF-Ox composites remains below that of the CF-Pr based ones, although they close in to each other at longer reaction times as the presence of the fiber becomes of lesser importance by the dilution. The surface treatment has most likely led to more anchor points for MgCl_2_ crystallite formation, and more catalyst precursor was consumed (Scheme 1), leading to locally lower polyethylene segment concentrations during polymer formation and, thus, a lower rate of crystallization in the early phase (CF-Ox 15 wt%). With that, larger crystal lamellae with a higher melting point form, and a larger amorphous part results as well (vide supra). The preparation of CF-Ox composites with a 5 wt% fiber content needs more time than for composites of CF-Pr because of the lower activity (Table 2). A fraction of higher-melting, larger lamellae forms in the later stage which shifts the melting point of the ever-broader DSC trace to higher values (Figure S3).

The crystallization temperature of the (virgin) melts tends to increase with the amount of fiber, explainable by a nucleating effect of either the CF or the MgCl_2_ support residues (Table 2, Figure S4a). The *T*_m_ of the compression-molded composites (Table 2, Figure S4b) tends to be higher and closer together in the range of 145 to almost 148 °C. After the polymerization process history is erased, the melting points are in a similar range for the similar matrix polymers. Simple trends of *T*_m_, crystallinity, and molecular mass along the fiber content do not seem to exist for the in situ prepared composites after thermal processing (Figure S4b,c). Then, compounds with a similar melting point, but with various crystallinities, appear.

The shore D hardness, according to DIN EN ISO 868 [40], of the reference UHMWPE and its composites with CF-Pr is also the same within error. The same is found for in situ composites of CF-Ox, almost at the same level. Apparently, the crystallinity and the presence of fibers do not change the hardness to a larger extent in those cases. The only substantial harder material is the in situ composite with 15 wt% CF-Pr (Table 2, Figure S4d). The fiber content does not exert a discernible influence on the hardness of the melt-compounded material; however, the proportion of hard and brittle “domains” increased. The agglomeration of fiber clusters and the poorer distribution of fiber within the melt-compounded composite and CF-Ox composites might relate to the larger standard deviations.

### 3.4. Mechanical Properties

**Figure 6:**
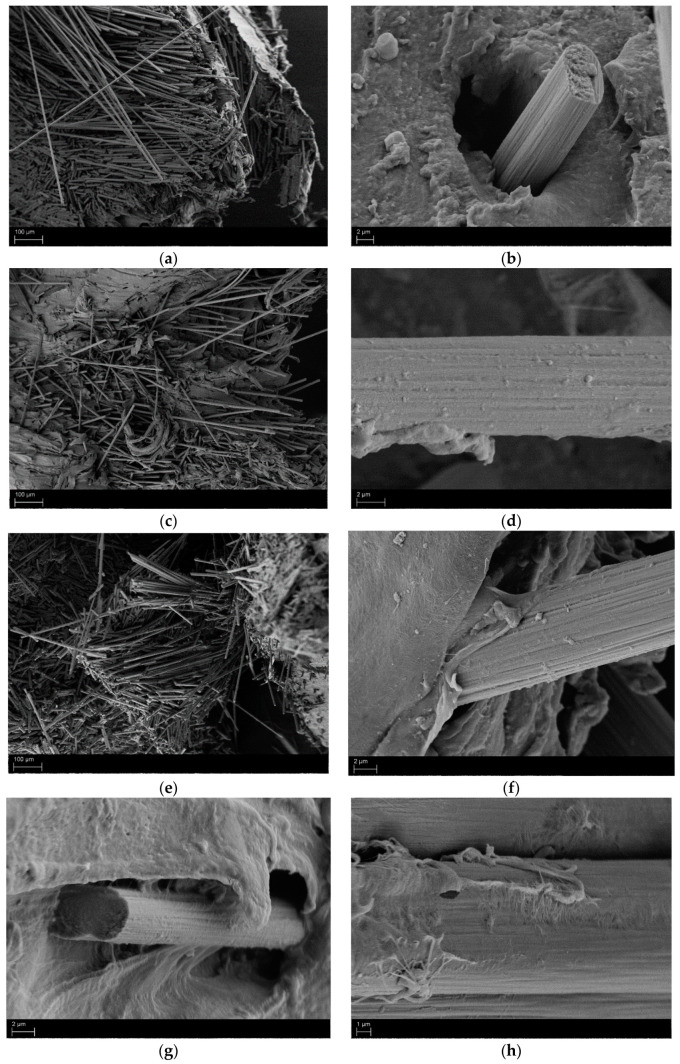
Fracture images of composites with 15 wt% of fibers: melt-compounded composites (**a**,**b**) in situ with CF-Pr (**c**,**d**), in situ with CF-Ox composites (**e**,**f**), and fibrils of interface of CF-Pr (**g**) and CF-Ox (**h**).

The mechanical properties of the composites are, of course, of most interest in this study. Testing bars of type 5A (DIN EN ISO 527-2 [38]) were cut from plates prepared by compression molding (190 °C, 200 bar pressure, and 10 min of holding time). Reference UHMWPE/CF composites (i.e., melt compound) were prepared by dry-mixing short-fiber CF-Pr with the reference UHMWPE, followed by compression molding into thin sheets, which were 10-fold stacked, with the package then again heat-compressed (190 °C, 200 bar, and holding time 1 min) to yield a plate of 2 mm thickness. The morphology of the composites after processing and after tensile force is applied is contrasting with the impression of single fibers after the synthesis (Figure S2): agglomerates of fibers/fiber bundles are now visible after compression (Figure 6a,c,e). They may have been (partly) reunited during compression molding in the case of the in situ composites, with the intermediate polyethylene particles pressed away from the fiber (before melting). The compatibility between the matrix and fibers is low in any case, particularly in the case of the more polar CF-Ox (Figure S5), and a driving force for separation may exist. The presence of agglomerates is more obvious at a higher fiber content, which may be expected anyway, and also on account of the polymerization outcome with the polymer formed next to and not such much inside the fiber bundles.

The tensile tests were used as a base case to evaluate mechanical performance. Important long-term behavior, like fatigue, and time-dependent mechanical responses [65,66], which are critical to evaluating the durability and real-world applications of the composites, are not included and should be addressed in the future. Tensile testing according to DIN EN ISO 527 [38,39] at a rate of 5 mm/min showed the behavior of pseudo-plastic materials with a yield point, cold flow, and strain hardening to break in the majority of measurements (Figure S6). The matrix experiences three-dimensional stress during uniaxial tension in the direction of the fibers, which is accompanied by the compression of the matrix around the perimeter of the fibers. Additional friction enhances the tensile strength as long as contact is maintained, leading to the ultimate cohesive matrix fracture or adhesive crack on the fiber surface. PAN-based carbon fibers themselves break at an elongation of only 0.4–1.2% [16], indicating that, at the observed elongation before the break of more than 10%, cohesive matrix failure is the dominant process in all cases. Indeed, SEM images of the fractures of the melt-molded compound shows a clear separation of the fiber and matrix with little evidence for mutual affinities (Figure 6a,b).

The stiffness in terms of the Young’s modulus increases monotonously with the content of the unmodified CF-Pr fibers of the melt-compounded and in situ composites (Table 3, Figure S7a). The in situ prepared composite based on CF-Pr with a 15 wt% of fiber content in UHMWPE has the highest Young’s modulus of over 3 GPa, almost an order of magnitude higher than that of the reference UHMWPE.

The Young’s moduli of the respective in situ CF-Pr composites are about twice as high as the melt-compounded analogs. One difference between the composites with CF-Pr is the dispersion of the fibers. The enhanced fiber dispersion increases the matrix–fiber contacts, enabling a more effective load transfer between the fibers and the matrix, thereby leading to a notable enhancement in stiffness [16,67,68]. The fibers in the reference UWMWPEs are not as nearly as well distributed as those of the in situ composite, leading to fewer matrix–fiber contacts (Figure 6a,c) and more non-load-carrying fiber–fiber contacts. The coarser distribution also explains the higher ductility at a lower fiber-loading of 5 and 10 wt% of the melt-molded composites with the higher volume of the continuous matrix phase. The latter seems to be lost at higher fiber contents, making the melt-compounded composite break at lower elongations.

A second difference between the CF-Pr-based composites is in the dependence of the yield strength and final tensile strength on the fiber content (Figure S7b,c). The mechanics of the reference UHMWPE composites are reminiscent of composites with little affinity between the fiber and matrix (Table 3). The yield stress is more or less that of the matrix (somewhat lower at higher loadings with less volume of polymer). It is further supported by the lower tensile strength at break.

In contrast, the yield strength of the in situ CF-Pr composites increase with the filler content, indicative of positive interactions between the matrix and the stiff fiber. The stress at yield tops those of the melt-compounded composites at any composition, with only the composite with 5 wt% of CF-Pr becoming quite similar to the reference composite (Figure S7b). This behavior is attributed to the presence of a mechanical interlocking between the fiber and matrix polymer, which appears to be of higher importance for the mechanical properties than, for example, the surface binding of the UHMWPE alone. The close association of the polyethylene and the carbon fibers could result in enhanced mechanical interlocking at the interface. Such an interlocking increases the tension required for a fiber pull-out, thus improving the load transfer between the matrix and the fiber. Consequently, a higher tensile strength at break is found in CF-Pr in comparison to standard melt-compounded composites (Table 3, Figure S7c). SEM images of the fracture surface give evidence of fibril formation, where the extension of the UHMWPE has taken place (Figure 6g). The mechanical interaction in the CF-Pr in situ composites between the fiber and matrix makes them also less ductile. The impression of the fracture surfaces is reminiscent of the SEM images of the virgin composites: bare (polyethylene/catalyst component covered) fibers occur with spots of imbedding in polyethylene crystallites (Figure 4e and Figure 6d).

The high value of the Young’s modulus of the in situ composites with 15 wt% of CF-Pr are not reached with the CF-Ox fibers, and there is no trend in the yield stress with the fiber content (Table 3). The CF-Ox composites are more ductile at higher loadings, indicative of a medium to low dispersion of the individual fibers (Figure S6). SEM images of the fracture surface of the composite with 15 wt% of CF-Ox also gives the impression of fiber bundles without the intercalation of matrix polymer (Figure 6e and Figure S5c), like the situation in the corresponding melt-compounded sample (contrasting the impression after synthesis in Figure S2f). The mechanical behavior is also more like the reference compound, reminiscent of the polymer-forming process, with an apparent catalyst system that is not as closely associated with the fiber (separating from the surface during polymer formation). At the same time, some polyethylene is found at the surface of the fibers (Figure 4b,d,f and Figure 6h). The formation of fibrils during extension, however, seems not as pronounced as in the case of CF-Pr in situ composites. Possibly, the interaction with the matrix is not as effective: the exposed fibers reveal a deboning within the matrix (Figure 6f).

The stiffness of the CF-Ox-based composites at 5 wt% of filler content in contrast are comparable in stiffness and ductility to those of the CF-Pr-based samples (both of the reference and in situ prepared samples), but the yield stress and tensile strength are appreciably higher (Table 3). The difference originates possibly from the strength of the established fiber–fiber interactions, which can be stronger for more polar surfaces. As the polymer content increases during polymerization, clusters of fibers tend to be resolved, increasing the matrix-filler contacts. This process of separation and possibly reagglomeration during the processing of the individual filler strains is, in the same sense, also dependent on the strength of the fiber–fiber interactions. Fiber clusters effectively (like in the melt-compounded samples) comprise more non-load-carrying filler–filler contacts, which create zones of stress concentration as points of weakness [69,70], but, at a lower concentration of agglomerates, also enhance the ductility.

The CF-Ox composite with a fiber content of 10 wt% exhibited the highest breaking strength and the largest elongation of the composites with a 10 wt% fiber, with not a lot of difference compare to the CF-Ox composite with 5 wt% of fibers (Figures S6a,b and S7c,d). This is again related to the higher polarity of the more oxidized carbon fibers with their higher interfacial energy, not having such an extensive separation, and possibly with a few interlocking points to the matrix (Figure 6e). This may underlie the stagnant reinforcing behavior of the composites based on CF-Ox at slightly above 2 GPa at 10 wt% and 15 wt%. The oxidation process is also a destructive process causing more defects on the fiber [71]. The rougher surface of the fibers may impede mechanical separation without strengthening the matrix–fiber interface (Figure 6f). The stronger fiber–fiber interaction promotes cluster formation. Thus, at 5 wt% of CF-Ox loading, the composite has a higher Young’s modulus and yield stress, and a ductility similar to the composites with 5 wt% of CF-Pr (Table 3).

## 4. Conclusions

Composites of UHMWPE with uniformly distributed fiber and fiber bundles were prepared by a simple procedure: a Ziegler–Natta catalyst system was created on and closely associated to the carbon fibers; subsequently, ethylene was polymerized between the fibers and fiber bundles. This was achieved by the treatment of CF-Pr and CF-Ox fibers with Mg(C_4_H_9_)_2_, AlEt_2_Cl, and TiCl_4_ with the result of a finely dispersed, polymerization-active species on and close to the fiber surface. The lower activity of the catalyst system in the presence of fibers suggests that parts of the catalyst system reacted to give a covalent bond to oxygen-containing entities at the fiber surface, which act at least as initial anchoring points. The fibers’ presence altered the catalyst’s kinetic behavior during polymerization, producing polymer chains with higher molar weights. The product morphology, the thermal properties, and the molecular weight (distribution) give a consistent view of the initial polymer formation near the fibers with the fragmentation of the catalyst in due course into the immediately surrounding solvent.

Both the stiffness and the yield strength are convincingly higher for the in situ prepared composites. Fiber pull-out with residual matrix contacts remained a significant weakness. Increasing the polarity of the fibers by oxidation increases the strength of fiber–fiber contacts but seems to decrease the wetting of the matrix. This has a negative effect on both the catalyst activity and the mechanics. The linear increase in the Young’s modulus of the CF-Pr composite is not found for CF-Ox composites and the elongation at break remains higher for the latter. The counteracting effects lead to superior composites at a 10 wt% content of fibers for CF-Ox. The highest stiffness and yield strength have the in situ composite with 15 wt% of CF-Pr fibers, with the best mechanical interlocking between the matrix and filler, which comes at the price of the lowest ductility.

## Figures and Tables

**Table 1:** SEM-EDX surface analysis of carbon fiber (CF-Pr) and oxidized carbon fiber (CF-Ox).

Exp.	Sample	O (wt%)	Mg (wt%)	Al (wt%)	Ti (wt%)
1	CF-Pr neat	3.48 ± 0.24	-	-	-
2	CF-Ox neat	7.41 ± 0.03	-	-	-
3	CF-Pr/cat ^a^	6.04 ± 0.91	0.17 ± 0.09	0.10 ± 0.06	0.03 ± 0.02
4	CF-Ox/cat ^a^	7.36 ± 0.74	0.23 ± 0.02	0.11 ± 0.02	0.03 ± 0.01

^a^: Mg(C_4_H_9_)_2_ (1 mmol/g_CF_), 30 min of impregnation time; AlEt_2_Cl ([Al]:[Mg] = 6) and TiCl_4_ ([Mg]:[Ti] = 500), 30 min for reaction at 50 °C.

**Table 2:** Data on reference UHMWPE and its composites with CF-Pr and CF-Ox.

Sample	Fiber Content (wt%)	Catalyst Activity (t_PE_/mol_Ti_·h)	Chains per Ti ^a^	*M*_W_ ^b^ (10^6^ g/mol)	PDI	*T*_m_ ^c^ (°C)	*T*_c_ ^c^ (°C)	*T*_m_ ^d^ (°C)	*X*_c_ ^d^ (%)	Shore Hardness (D)
UHMWPE	-	7.0	3	5.1	6.5	140.4	105.5	145.4	68	67 ± 1
Melt compound	5	-	-	-	-	-	-	147.7	55	68 ± 2
	10	-	-	-	-	-	-	146.9	54	66 ± 2
	15	-	-	-	-	-	-	145.1	46	67 ± 2
CF-Pr	5	6.5	2.8 ± 0.2	6.5 ± 0.0	3.8 ± 0.2	145.4	108.2	146.5	43	69 ± 1
	10	6.8	2.3 ± 0.4	5.4 ± 0.3	4.9 ± 0.6	144.9	109.4	147.5	53	70 ± 1
	15	6.9	0.7 ± 0.1	5.3 ± 0.1	2.4 ± 0.2	143.1	110.0	145.5	46	76 ± 1
CF-Ox	5	5.6	2.8 ± 0.2	5.9 ± 0.0	3.5 ± 0.2	148.5	107.8	147.8	46	72 ± 4
	10	6.1	2.2 ± 0.4	5.4 ± 0.3	5.0 ± 0.9	144.8	111.3	146.5	44	72 ± 2
	15	6.7	1.3 ± 0.4	4.7 ± 0.6	4.6 ± 1.4	144.6	109.9	146.1	46	71 ± 3

^a^: Polymer chains per Ti was calculated by using product yield per the number-average molecular weight *M*_n_ of the resulting polymer and total amount of Ti, ^b^: from SEC against PS standards, ^c^: first heating/cooling scan of maiden composites, ^d^: first heating scan of compression-molded composites.

**Table 3:** Mechanical properties on reference UHMWPE and composites with CF-Pr and CF-Ox.

Sample	Fiber Content (wt%)	Y-M ^a^ (GPa)	*σ*_yield_ ^b^ (MPa)	*σ*_break_ ^c^ (MPa)	*ε*_break_ ^d^ (%)
UHMWPE	-	0.36 ± 0.02	26.1 ± 0.2	34.4 ± 1.9	285 ± 8
Melt compound	5	0.76 ± 0.05	30.4 ± 0.1	20 ± 5.8	107 ± 15
	10	1.04 ± 0.05	32.0 ± 1.6	15 ± 5.7	43 ± 7
	15	1.60 ± 0.17	28.0 ± 2.6	6.4 ± 1.0	8 ± 1
CF-Pr	5	1.54 ± 0.03	32.4 ± 0.5	20 ± 9.4	80 ± 13
	10	2.24 ± 0.04	41.6 ± 0.7	23 ± 6.9	23 ± 4
	15	3.20 ± 0.10	50.0 ± 1.3	15 ± 4.9	12 ± 1
CF-Ox	5	1.79 ± 0.04	39.1 ± 0.6	28 ± 1.4	75 ± 17
	10	2.19 ± 0.07	38.4 ± 0.6	34 ± 5.5	85 ± 13
	15	2.10 ± 0.09	33.0 ± 1.6	22 ± 5.4	40 ± 14

^a^: Young’s modulus, ^b^: Yield stress, ^c^: Tensile strength at break, ^d^: Elongation at break.

## Data Availability

The data are contained within the article or Supplementary Materials.

## Supplementary Materials

The following supporting information can be downloaded at: https://www.mdpi.com/article/10.3390/polym17010090/s1, Figure S1: Catalyst activity depending on fiber content for reference UHMWPE, UHMWPE/CF-Pr, and UHMWPE/CF-Ox; Figure S2: SEM images of overview of maiden CF-Pr after in situ polymerization at fiber content of 5 wt% (a), 10 wt% (c), and 15 wt% (e), and virgin CF-Ox composites with 5 wt% (b), 10 wt% (d), and 15 wt% (f) fiber content; Figure S3: Melting behaviors of reference UHMPWE and the in situ prepared composites with CF-Pr (blue) and CF-Ox (red) at the first heating scan; Figure S4: *T*_c_ of the first heating scan of virgin composites (a), *T*_m_ (b), the degree of crystallization (c), and Shore D hardness (d) of reference UHMWPE, melt-compounded material, CF-Pr, and CF-Ox composite samples after compression molding; Figure S5: Fracture images of CF-Pr composite at fiber content of 5 wt% (a) and 15 wt% (b) and CF-Ox composite at 5 wt% (c) and 15 wt% (d) fiber content; Figure S6: Tensile diagram of neat reference UHMWPE materials, melt-compounded composite, and CF-Pr and CF-Ox composites with 5 wt% (a), 10 wt% (b), and 15 wt% (c) fiber content; Figure S7: Young’s modulus (a), yield strength (b), tensile strength at break (c), and tensile strain at break (d) of reference UHMWPE, melt-compounded material, and CF-Pr and CF-Ox composites.
