# Supplementary material for: In Situ Polymerization and Synthesis of UHMWPE/Carbon Fiber Composites
Source: Polymers (Basel). 2025 Jan 1;17(1):90. doi: 10.3390/polym17010090 (PMC11722603; doi:10.3390/polym17010090)
Supplement: Supplementary file 1 [file polymers-17-00090-s001.zip › polymers-3354954-supplementary.pdf]

Supplementary Material belonging to

In-situ Polymerization and Synthesis of UHMWPE/ Carbon Fiber-Composites

Elena Fedorenko and Gerrit A. Luinstra*

Institute for Technical and Macromolecular Chemistry, University of Hamburg, Bundesstraße 45, 20146 Hamburg, Germany; elena.fedorenko@uni-hamburg.de

* Correspondence: Luinstra@chemie.uni-hamburg.de; Tel.: +49 40 42838-3162

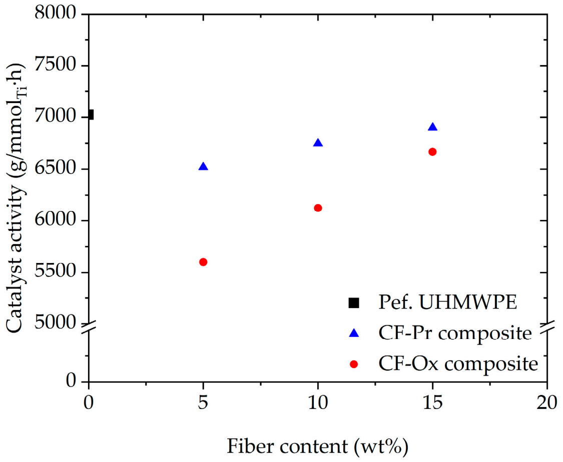

Figure S1. Catalyst activity depending on fiber content for reference UHMWPE, UHMWPE/CF-Pr and UHMWPE/CF-Ox.

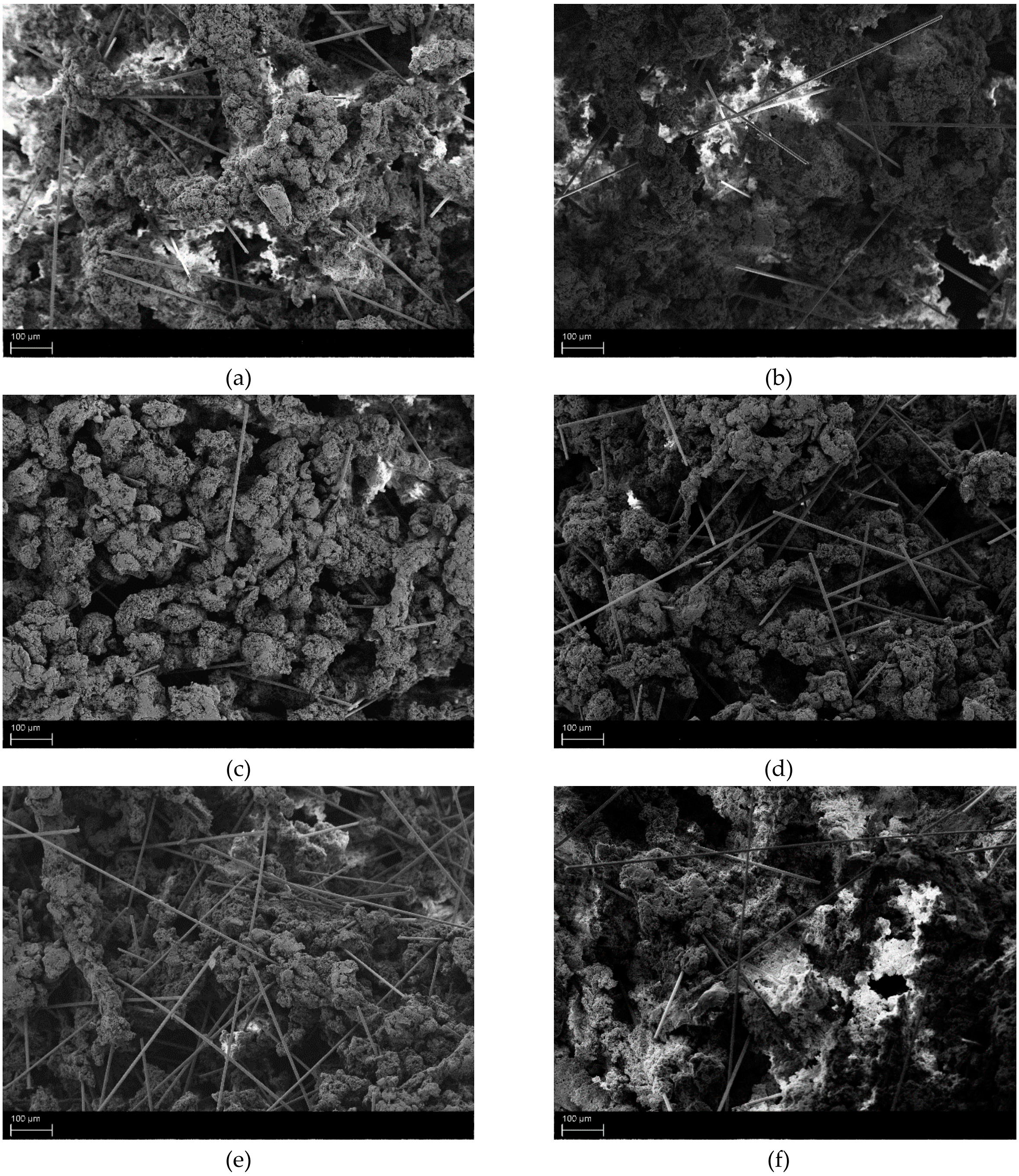

Figure S2. SEM images of maiden CF-Pr after in situ polymerization at fiber content of 5 wt% (a), 10 wt% (c) and 15 wt% (e), and virgin CF-Ox composites with 5 wt% (b), 10 wt% (d) and 15 wt% (f) fiber content. Images are of easily gained particle fragments of the polymerisate, and are not representative for the sample (c.f. **Figure S5**).

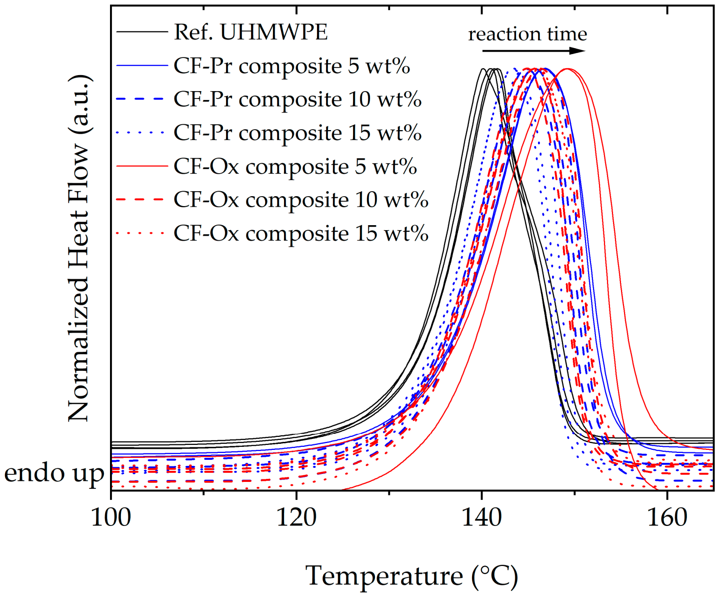

Figure S3. Melting behavior of reference UHMPWE and the in-situ prepared composites with CF-Pr (blue) and CF-Ox (red) at the first heating scan.

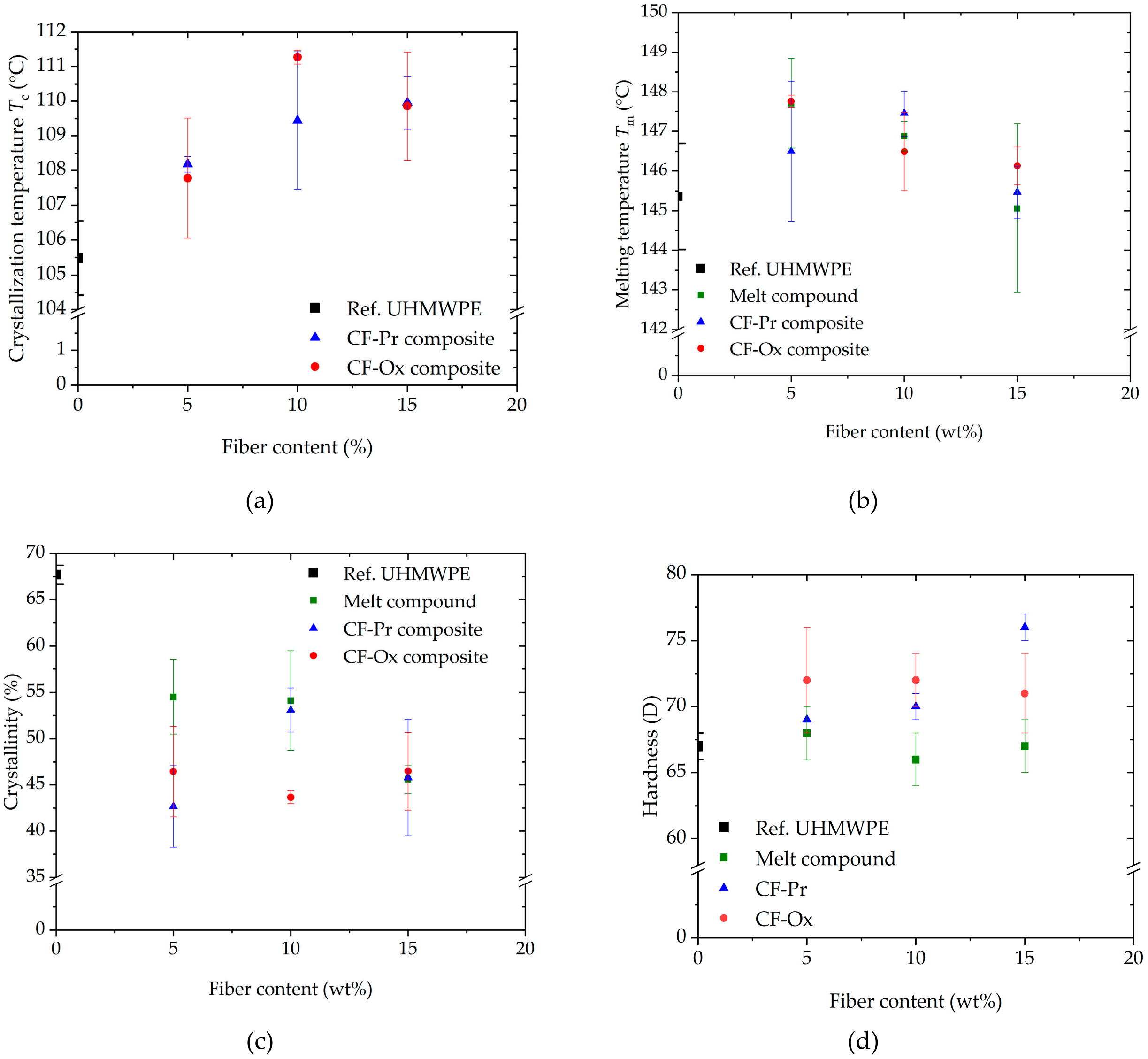

Figure S4. T_c of the first heating scan of virgin composites (a), T_m (b), the degree of crystallization (c) and Shore D hardness (d) of reference UHMWPE, melt compounded material, CF-Pr and CF-Ox composite samples after compression molding.

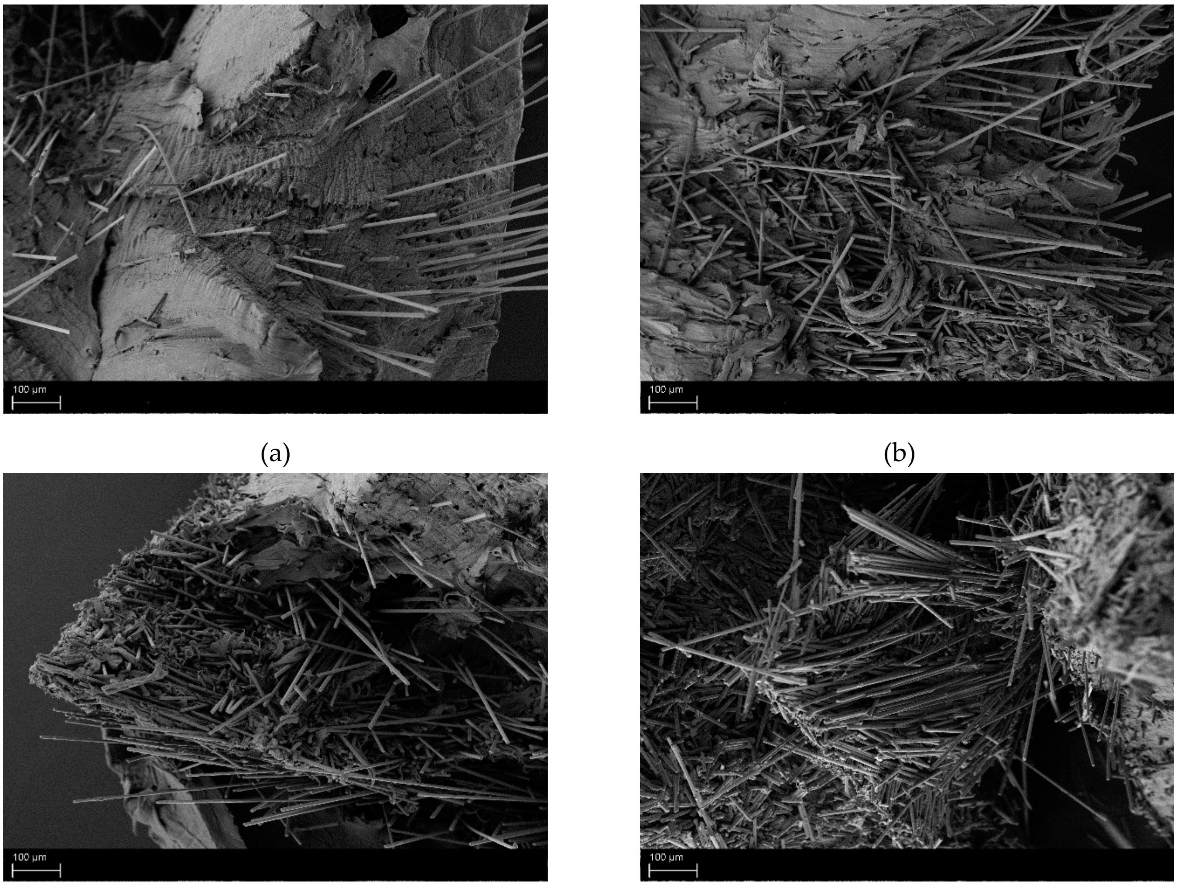

(a) (b) (c) (d)

Figure S5. Fracture images of CF-Pr composite at fiber content of (a) 5 wt% and (b) 15 wt% and (c) CF-Ox composite at 5 wt% and (d) 15 wt% fiber content.

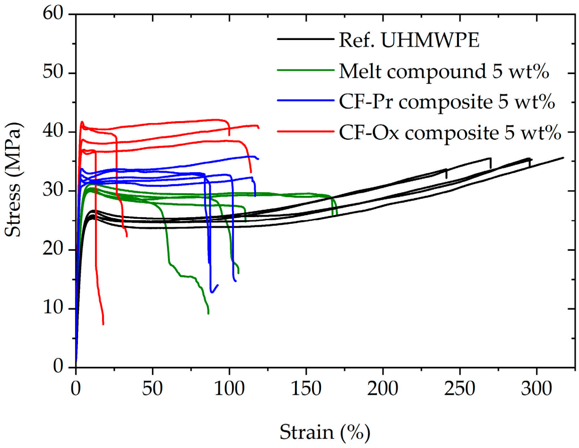

(a)

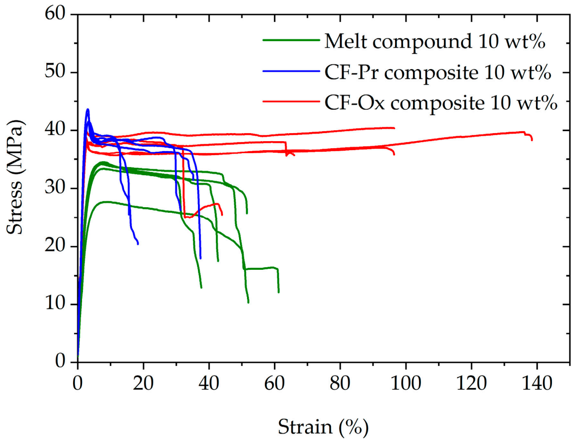

(b)

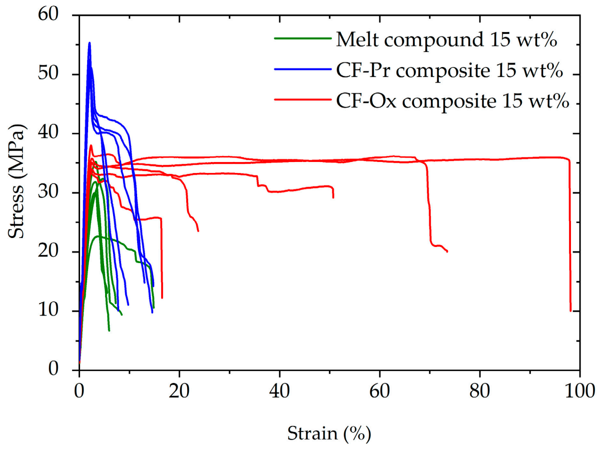

(c)

Figure S6. Tensile diagram of neat reference UHMWPE materials, melt compounded composite, CF-Pr and CF-Ox composites with (a) 5 wt%, (b) 10 wt% and (c) 15 wt% fiber content.

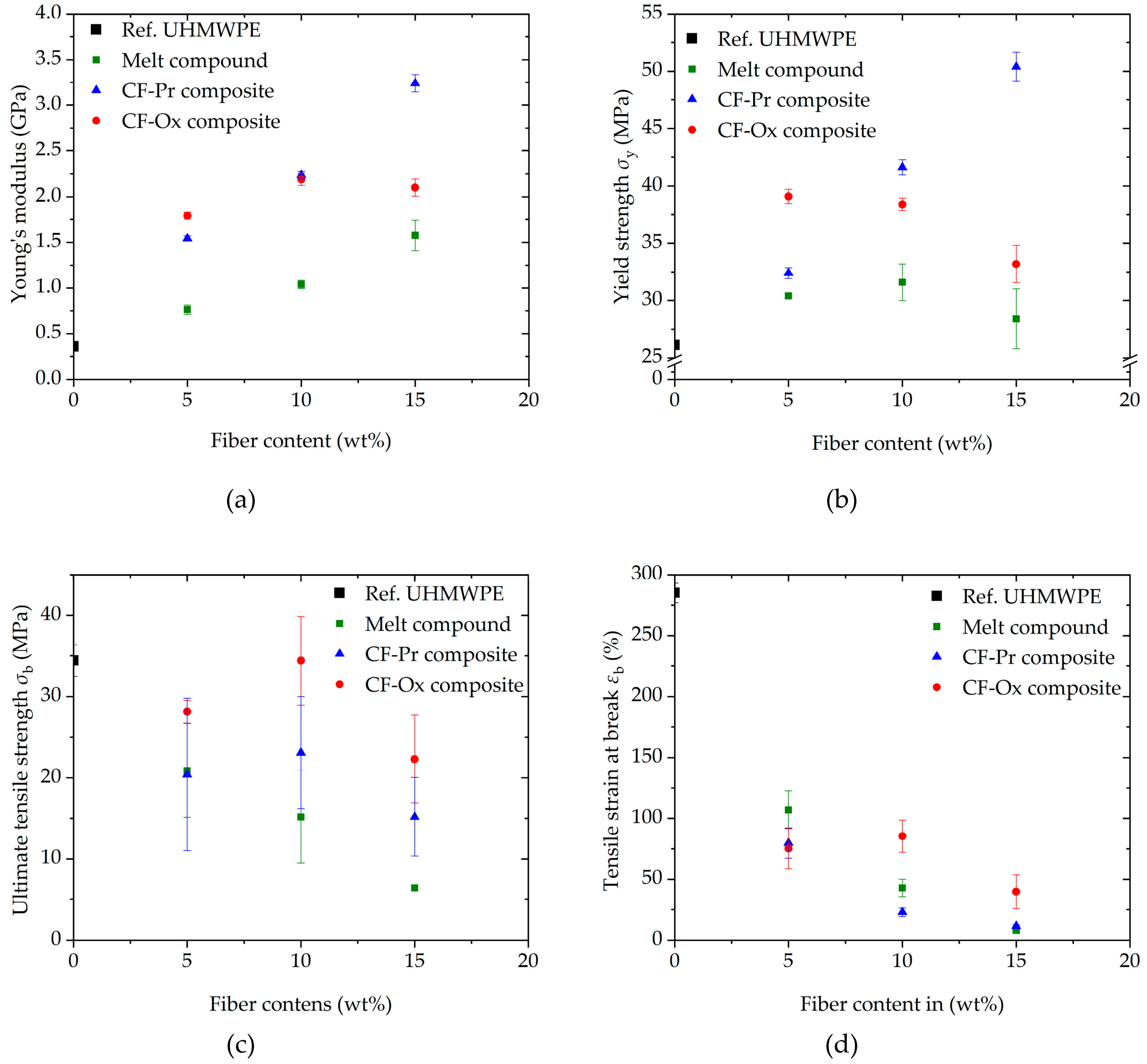

Figure S7. Young´s modulus (a), yield strength (b), tensile strength at break (c) and tensile strain at break (d) of reference UHMWPE, melt compounded material, CF-Pr and CF-Ox composites.
